# Extreme Behaviors in Fibrous Material Remodeling: Auxetic to Non-Auxetic Transition and Phase Segregation

**DOI:** 10.3390/ma18071674

**Published:** 2025-04-06

**Authors:** Andrea Rodella

**Affiliations:** Department of Structural and Geotechnical Engineering, Sapienza University of Rome, Via Eudossiana 18, 00185 Rome, Italy; andrea.rodella@uniroma1.it

**Keywords:** fibrous material, auxetic, segregation, remodeling, plasticity, variational approach

## Abstract

Fibrous materials, prevalent in biological tissues and engineered composites, undergo remodeling in response to mechanical loads, leading to plastic changes in fiber orientation. A previously developed continuum model describes this remodeling process. Building on that framework, the present study examines the extreme behaviors of such materials. Analytical results for the homogeneous response under tensile loading reveal three distinct classes: in class (A), fibers asymptotically approach a specific angle; in class (B), fibers align perpendicularly to the load direction; and in class (C), fibers align either with the load direction or perpendicularly, depending on their initial orientation. Numerical simulations are employed to analyze the non-homogeneous material response in a standard tensile test, demonstrating how differences in behavior arise from the material class and the initial fiber orientation distribution. This investigation focuses on the extreme behaviors of material classes (A) and (C), emphasizing phase segregation and transitions between auxetic and non-auxetic behavior.

## 1. Introduction

Fibrous materials have recently garnered significant interest due to their essential roles in both biological systems and engineered materials. In biological contexts, human tissues and organs, such as skin, cartilage, and connective tissues, are structured with microsized fibers, which are crucial for maintaining mechanical integrity and functionality. Similarly, engineered fibrous materials, including textiles, nonwovens, and composites, are designed with similar architectures and are extensively studied for their unique mechanical properties and diverse applications [1,2,3,4].

Fibrous materials, both in biological systems and engineered materials, can undergo plastic reorganization under mechanical loading, leading to changes in fiber orientation. This remodeling process is especially relevant in biological tissues, where mechanical stimuli at the chemo-mechanical level drive the reorganization of collagen fibers. For instance, in the arterial wall, stress-driven collagen fiber remodeling plays a significant role in adapting the tissue to varying loads and pressures [5,6]. The reorientation of fibers in response to stress is a key mechanism for maintaining the tissue structural integrity and functionality, as seen in blood vessels where collagen fibers remodel to accommodate changes in mechanical demands [7,8]. Similarly, in soft tissues such as skin and cartilage, the alignment and reorganization of fibers influence the mechanical properties and adaptability of the tissues, contributing to their ability to withstand dynamic loads [9,10].

These processes are not limited to biological tissues; engineered materials, including hydrogels and fiber-reinforced composites, also exhibit fiber reorientation under mechanical loading. For example, studies have shown that the alignment of fibers in hydrogel-based materials can significantly affect their mechanical properties, such as stiffness and elasticity [11,12]. Moreover, understanding these remodeling processes has direct applications in designing materials for soft robotics and tissue engineering, where the ability to control and predict fiber orientation is crucial for optimizing material performance [13,14].

The mechanical properties of these tissues include both elastic [15,16] and inelastic [17,18,19] responses. Specifically, the extracellular matrix can undergo plastic remodeling, leading to irreversible deformations that significantly affect cell behavior, such as migration and differentiation [19,20,21].

The effects of reorientation, particularly post-yield deformation, remain largely unexplored in both biological and engineered composites. This study focuses on the plastic threshold mechanism, neglecting viscous effects and other secondary properties. As a result, the asymptotic behavior is determined solely by the choice of free energy, allowing for analytical solutions in two-dimensional (2D) problems [22]. The model is grounded in the principles of continuum mechanics, specifically developed for materials incorporating an internal state variable [23,24,25,26,27]. The model introduced in [22] describes the behavior of fiber reorientation within these materials and demonstrates that remodeling, defined as the reorganization of the internal structure, induces diverse material responses.

In particular, when the analysis is restricted to a 2D beam subjected to traction, the fibers within the material can align themselves in different ways depending on their initial orientation. The fibers may eventually align at a specific angle, perpendicularly to the direction of the applied load, or along the load direction.

Notably, fiber reorganization can induce substantial changes in mechanical behavior, including transitions between auxetic and non-auxetic responses, as well as the emergence of regions with distinct mechanical properties due to phase segregation. While previous studies have established the role of fiber reorientation in influencing material properties, a comprehensive understanding of how these transitions emerge, the conditions under which they occur, and their structural consequences remains incomplete.

The paper addresses this gap by building on the continuum model presented in [22] to investigate the underlying mechanisms driving these phase transitions. By analyzing how fiber realignment governs the macroscopic response of the material, this study provides new insights into the tunability of fibrous materials, with long-term implications for applications in both biological systems and engineered structures.

The paper is organized as follows: Section 2 introduces the continuum model proposed in [22], outlining the key assumptions related to (i) the free energy governing the elastic response and (ii) the plastic reorientation model that describes the dissipation process, defined by the yield function. Section 3 summarizes the main analytical results from [22], focusing on a traction problem in the homogeneous case. Section 4 describes the numerical methods used to obtain the results presented in Section 5, which illustrate phase segregation and the transition from auxetic to non-auxetic behavior. Section 6 discusses how these results can be applied to real-world scenarios. Finally, Section 7 offers concluding remarks and outlines directions for future research.

## 2. Free Energy and Reversibility Domain Assumptions

Consider a 2D body B where each point *x* has a position vector $x=x_{\alpha }e_{\alpha }$ relative to the origin *o*. Each point is associated with an oriented fiber, whose direction is given by the unit vector $n\left(\vartheta \right)=\cos\vartheta e_{1}+\sin\vartheta e_{2}$, where ϑ is an internal state variable, see Table 1 for the notation. Following the theories [23,24,25], ϑ influences the free energy, and its evolution dictates dissipation.

The state of B at time *t* is defined by Λ={E,ϑ}, where E=sym∇u is the linearized strain based on the displacement field u. The free energy density is a quadratic function of the strain:

(1) $$\psi =\hat{\psi }\left(E,\vartheta \right)=\frac{1}{2}C\left(n(\vartheta )\right)E\cdot E.$$

Here, $C\left(n(\vartheta )\right)$ is the elasticity tensor for a transversely isotropic material with respect to n(ϑ). In 2D, the free energy density is as follows:

(2) $$\hat{\psi }\left(E,\vartheta \right)=\mu {∥E∥}^{2}+\frac{\lambda }{2}{\left(\mathrm{tr}E\right)}^{2}+c_{1}\left(\mathrm{tr}E\right)\;E\;n\left(\vartheta \right)\cdot n\left(\vartheta \right)+c_{2}\;{\left(E\;n(\vartheta )\cdot n(\vartheta )\right)}^{2},$$

where λ and μ are the Lamé coefficients, and $c_{1}$ and $c_{2}$ characterize the transverse isotropy. With fixed Lamé coefficients (μ>0, 2μ+λ>0), the pair $\left(c_{1},c_{2}\right)$ must belong to a set P to ensure the positiveness of the energy density:

(3) $$P=\left\{\left(c_{1},c_{2}\right)\in R:\;c_{2}>\frac{c_{1}^{2}-4\mu c_{1}-4\mu \left(\mu +\lambda \right)}{2(2\mu +\lambda )}\right\}.$$

**Figure 1:**
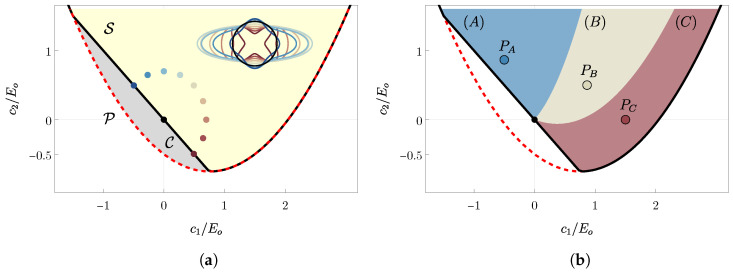
(**a**) The red dashed line confines the set where $\left\{c_{1},c_{2}\right\}/E_{0}$ ensures positive energy (Equation (3)), with $E_{0}$ being the isotropic Young modulus. The yellow area, a subset of P, represents the stiffening set (Equation (5)). The insert shows polar plots of Young modulus $\hat{E}\left(\alpha ,\vartheta =0\right)$ for α∈[0,2π], corresponding to colored dots from the stiffening set S. (**b**) Colored areas depict asymptotic behaviors of fiber orientation ϑ in traction tests (see [22]): alignment at a given angle (blue), perpendicular alignment to the load (gray), and alignment either perpendicularly or with the load, depending on initial orientation (red).

This set is represented by the red dashed parabola plotted in Figure 1. To understand the role of $c_{1}$ and $c_{2}$, the author recall the Young modulus and Poisson ratio definitions for an uniaxial traction test $\bar{T}=\sigma t⊗t$ in the direction $t=\cos\alpha e_{1}+\sin\alpha e_{2}$. The corresponding strain is $\bar{E}=C^{-1}\left(n(\vartheta )\right)\bar{T}$. Thus, Young modulus and Poisson ratio depend on the angle (α−ϑ) between the testing direction and the fiber orientation:

(4) $$E\left(\alpha ,\vartheta \right)=\hat{E}\left(\alpha ,\vartheta \right):=\frac{\bar{T}t\cdot t}{\bar{E}t\cdot t};\;\nu \left(\alpha ,\vartheta \right)=\hat{\nu }\left(\alpha ,\vartheta \right):=\frac{\bar{E}t^{⊥}\cdot t^{⊥}}{\bar{E}t\cdot t}.$$

Polar plots illustrate how Young modulus varies with (α−ϑ), as shown in the insert of Figure 1a. The stiffening set S is defined by ensuring that the ratio of Young moduli parallel and perpendicular to the fiber direction ($E_{\|}=\hat{E}\left(\alpha =\vartheta ,\vartheta \right)$ and $E_{⊥}=\hat{E}\left(\alpha =\vartheta -\pi /2,\vartheta \right)$) is greater than 1 as follows:

(5) $$\frac{E_{\|}}{E_{⊥}}=1+\frac{2(c_{1}+c_{2})}{2\mu +\lambda }>1\;⟹\;S=\left\{\left(c_{1},c_{2}\right)\in P:\;c_{1}+c_{2}>0\right\}.$$

This set is represented by the yellow area in Figure 1a. Conversely, the compliance set C=P∖S represents materials where fibers reduce stiffness in their orientation direction. This set is depicted in gray in Figure 1a. For more details on the possible application of this set, the reader is referred to [28,29], where the authors discuss the potential use of these materials in modeling the tethering of fibers in the extracellular matrix of biological tissues.

Within the Generalized Standard Materials framework [25], dissipation is described by the dissipation potential, a convex and positive function of the internal state variable rates. Since n·n=1 and $\dot{n}=\beta \;n^{⊥}$ with $n^{⊥}:=\partial_{\vartheta }n\left(\vartheta \right)=-\sin\vartheta e_{1}+\cos\vartheta e_{2}$, then the scalar product between $\dot{n}\cdot n=0$. Thus, from Equation (2), the energy release rate takes the form:

(6) $$\begin{array}{cc} -\partial_{\vartheta }\hat{\psi }\left(E,\vartheta \right)\dot{\vartheta } & =-\partial_{n}\hat{\psi }\left(E,n\left(\vartheta \right)\right)\cdot \dot{n}\left(\vartheta \right)\\ \; & =-2\left(c_{1}\mathrm{tr}E+2c_{2}En\left(\vartheta \right)\cdot n\left(\vartheta \right)\right)En\left(\vartheta \right)\cdot \beta n^{⊥}\left(\vartheta \right)\\ \; & =-\beta \gamma (E,\vartheta ), \end{array}$$

where γ(E,ϑ) is the remodeling torque associated with changes in fiber orientation ϑ. To satisfy the fundamental inequality, [25], the energy release rate at E must exceed that at any admissible $\tilde{E}$:

(7) $$-\beta [\gamma \left(E,\vartheta \right)-\gamma \left(\tilde{E},\vartheta \right)]>0$$

for any E and $\tilde{E}$ in the reversibility domain, R(η). This domain comprises symmetric strain tensors for which the remodeling torque remains below a critical threshold η>0:

(8) $$R\left(\eta \right)=\left\{E\in \mathrm{Sym}\;:\;\underset{\vartheta \in [-\pi /2,+\pi /2]}{\sup}\left|\gamma \left(E,\vartheta \right)\right|\leq \eta \right\}.$$

This set represents strains for which the fiber orientation ϑ remains constant, resulting in purely elastic material behavior. Its boundary, named *yield set*, is denoted by $\partial R\left(\eta \right)=\{E:\sup_{\vartheta }|\gamma \left(E,\vartheta \right)|=\eta \}$. Following [25,30], the dissipation rate is given by the Legendre transform:

(9) $$d\left(\vartheta \right)\left(\dot{\vartheta }\right)=\underset{E\in R(\eta )}{\sup}\left(-\partial_{\vartheta }\hat{\psi }\left(E,\vartheta \right)\dot{\vartheta }\right)=\eta \;\beta \;|\dot{\vartheta }|=\bar{\eta }\left|\dot{\vartheta }\right|.$$

Considering a quasi-static process over a time interval *t*, the total dissipation is

(10) $$\delta =\hat{\delta }\left(t\right)=\int_{0}^{t}d\left(\vartheta \left(\tau \right)\right)\left(\dot{\vartheta }\left(\tau \right)\right)\;d\tau =\bar{\eta }\;\int_{0}^{t}\left|\dot{\vartheta }\left(\tau \right)\right|\;d\tau =:\bar{\eta }\;\bar{\Theta }\left(t\right),$$

where $\bar{\Theta }\left(t\right)$ is the accumulated fiber rotation over time *t*. Finally, the total energy is the integral of the free energy density, Equation (1), plus the remodeling dissipation, Equation (10), over the body B:

(11) $$E\left(E(t),\vartheta (t)\right)=\int_{B}\left(\hat{\psi }\left(E(t),\vartheta (t)\right)+\hat{\delta }\left(t\right)\right)\;dA.$$

## 3. Analytical Solution for the Homogeneous Material in a Traction Problem

**Figure 2:**
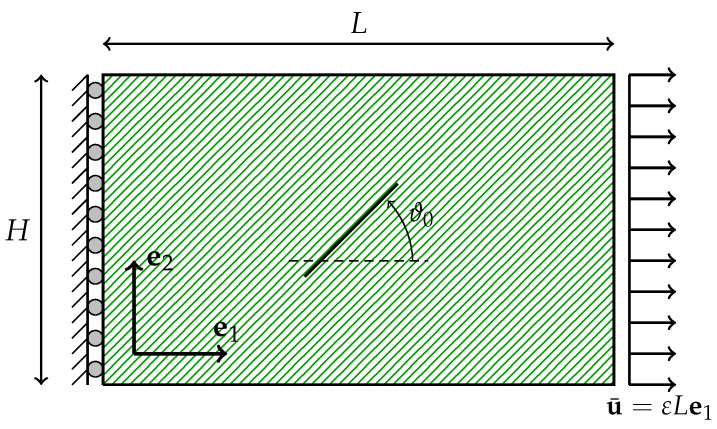
Sketch of the setup for the traction problem, where $\vartheta_{0}$ represents the initial orientation of the fibers.

Consider the traction problem sketched in Figure 2, where a rectangular sample of length *L* and height *H* is free on the upper and lower edges and allowed to slide on the left side. The points on the right side experience a horizontal displacement of $\bar{u}=\varepsilon L\;e_{1}$, whilst their vertical displacement remains unconstrained.

In [22], this problem is presented a complete analytical characterization of the homogeneous case in terms of strain and fiber rotation, as the imposed horizontal displacement $\bar{u}=\varepsilon \;Le_{1}$ monotonically increases starting from ε=0. The fiber orientation ϑ evolves according to the following Karush Kuhn Tacker (KKT) conditions [31]:

(12) $$\dot{\vartheta }\geq 0,\;\left|\gamma \left(E,\vartheta \right)\right|-\eta \leq 0,\;\dot{\vartheta }\left(|\gamma (E,\vartheta )|-\eta \right)=0.$$

Hence, it is possible to find an asymptotic value of the fiber orientation for ε→∞ once the material reaches the plastic regime:

(13) $$\vartheta_{\infty }\left(\lambda ,\mu ,c_{1},c_{2}\right)=\frac{1}{2}\arccos\left(\frac{2\mu (c_{1}+c_{2})}{c_{1}^{2}-2c_{2}\left(\mu +\lambda \right)}\right).$$

**Figure 3:**
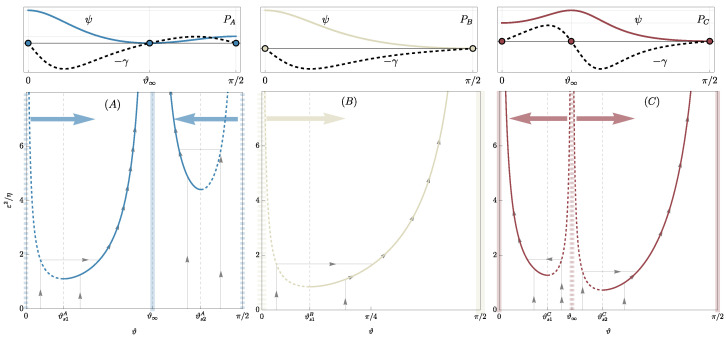
The top row illustrates the free energy landscape ψ (solid colored lines) and its derivative $\partial_{\vartheta }\psi =-\gamma$ (dashed black line) for material classes (A), (B), and (C), corresponding to points $P_{A}$, $P_{B}$, and $P_{C}$ in Figure 1b, distinguished by blue, white, and red colors, respectively. The bottom row depicts the evolution, in the yield set, of the fiber orientation ϑ from various initial orientations; continuous gray lines indicate the evolutionary paths, which can be continuous or exhibit jumps.

In summary, the stiffening materials are identified in three classes as represented in different colored regions in Figure 1b:

Class (A): Materials in class (A) exhibit a minimum of the free energy ψ at $0<\vartheta_{\infty }<\pi /2$ (it suffices to consider ϑ∈[0,π/2] as ϑ≡ϑ+π and, for the symmetry of the problem, ϑ≡−ϑ) and two local maxima at ϑ={0,π/2}. See, for instance, blue dots to the top left of Figure 3 for an illustration. The evolution of the homogeneous traction problem, starting from an initial uniform fiber orientation $\vartheta_{0}\in \left[0,\pi /2\right]$, is depicted in the bottom left of Figure 3. The fiber orientation evolves with jumps if $\vartheta_{0}$ lies on an unstable branch (dashed blue lines in Figure 3 bottom left, corresponding to $0\leq \vartheta_{0}<\vartheta_{s1}^{A}$ or $\vartheta_{s2}^{A}<\vartheta_{0}\leq \pi /2$), or continuously if $\vartheta_{0}$ is initially on a stable branch (continuous blue lines in Figure 3 bottom left, corresponding to $\vartheta_{s1}^{A}\leq \vartheta_{0}<\vartheta_{\infty }$ or $\vartheta_{\infty }<\vartheta_{0}\leq \vartheta_{s2}^{A}$). From a physical standpoint, materials in class (A) exhibit preferred fiber orientations that do not align with principal loading directions, leading to anisotropic stiffening effects that are particularly useful in biological tissues or engineered composites where directional reinforcement is required. For example, in muscle fibers or fibrous composite laminates, such energy landscapes help in tailoring mechanical responses to external forces while maintaining structural integrity[32,33].

Class (B): Materials in class (B) exhibit a single minimum of ψ at ϑ=π/2, with ϑ=0 representing a maximum. Furthermore, $\vartheta_{\infty }$ does not exist within the real numbers. Refer to the middle top of Figure 3 for the energy landscape and the middle bottom for the evolutionary paths of a generic initial fiber orientation, illustrated by continuous gray lines with arrows. Physically, class (B) materials favor alignment of fibers in a single dominant direction (ϑ=π/2), which suggests a material design where unidirectional reinforcement is beneficial. Such a configuration is particularly relevant in fiber-reinforced polymers and natural materials such as tendons, where maximizing stiffness along a specific axis is crucial for load-bearing efficiency and mechanical performance under tension.

Class (C): Materials in class (C) exhibit two minima of ψ at ϑ={0,π/2}, with $\vartheta_{\infty }$ corresponding to a maximum. Refer to the top right panel of Figure 3 for the energy landscape and the bottom right for the evolutionary paths of a generic initial fiber orientation, illustrated by continuous gray lines with arrows. As indicated by the large red arrows, $\vartheta_{\infty }$ is a critical point, meaning that a fiber initially oriented at $\vartheta_{\infty }+\alpha$, with α being a small perturbation, will reorient towards π/2. Conversely, if the initial orientation is $\vartheta_{\infty }-\alpha$, the final orientation will be 0. This scenario allows for the observation of phase segregation in a material with particular distributions of fiber orientation. In practical terms, class (C) materials exhibit bistable behavior, which is crucial for applications where controlled anisotropy and phase separation are desirable. This includes smart materials, such as shape-memory polymers or adaptive bio-inspired structures, where switching between two stable configurations under mechanical stimuli can be leveraged for tunable stiffness, actuation, or self-healing capabilities. Additionally, phase segregation effects in this class are particularly relevant for tissue engineering, where fiber alignment can dictate cellular growth patterns and mechanical functionality in biomimetic scaffolds [34,35].

By classifying materials based on these energy profiles, this framework provides a fundamental understanding of how the fiber orientation process influences mechanical behavior. This insight aids in designing materials with tailored anisotropic properties for various engineering and biomedical applications, ranging from high-performance composites to biomimetic tissues.

## 4. Numerical Implementation

The energetic properties of the problem solution and the general conditions offered by the incremental energy minimization framework [36] suggest the alternating minimization (AM) strategy as a natural resolution algorithm. In this specific case, the AM consists of two sequential minimization steps repeated over each time increment until convergence: first, the total energy is minimized with respect to the displacement field, u, while keeping the fiber rotational field, ϑ, fixed. Second, the total energy is minimized with respect to ϑ, while keeping u fixed. This process is detailed in the pseudo-code Algorithm A1.

The first step is equivalent to solving a linear elasticity problem. The second step is a nonlinear problem, but it is local in space, allowing for minimization via a local projection method, such as a modified Newton-Raphson scheme. The problem requires discretization in both time and space. The time discretization is merely a path-ordering parameter since no dynamics is involved.

Space Discretization: The domain $B=\{\left(x_{1},x_{2}\right):x_{1}\in \left(0,L\right)\wedge x_{2}\in \left(0,H\right)\}$ is discretized using an unstructured mesh. The displacement field u is approximated using a piece-wise affine finite element space [37] over B. The fiber rotational field ϑ, due to its intrinsic locality (a feature common in rate-independent plasticity problems), is projected onto a discontinuous finite element space over B.

Time Discretization: The time interval [0,T] is discretized in *n* non-uniform time steps $dt_{i}$. The accumulated fiber rotation is discretized as follows:

(14) $$\bar{\Theta }\left(t_{i}\right)≃{\bar{\Theta }}_{i}={\bar{\Theta }}_{i-1}+∥\vartheta_{i}+\vartheta_{i-1}∥.$$

At each time step *i*, the solution, representing the state Λ of the sample, must be a local minimum of the total energy with respect to both fields:

(15) $$\left\{u_{i},\vartheta_{i};{\bar{\Theta }}_{i}\right\}=\underset{u,\vartheta }{\mathrm{argmin}}\left\{E_{i}\left(u,\vartheta ;\bar{\Theta }\right)\right\},\;E_{i}\left(u,\vartheta ;\bar{\Theta }\right)=\int_{B}\psi_{i}\left(u,\vartheta \right)+\delta_{i}\left(\vartheta ;\bar{\Theta }\right),$$

subject to the Dirichlet boundary conditions $u\left(0,x_{2}\right)\cdot e_{1}=0$ and $u\left(L,x_{2}\right)\cdot e_{1}=\bar{u}\left(\varepsilon_{i}\right)$, where $E_{i}$ represents the time-discretized form of the total energy in Equation (11).

The code implementation uses python as an interface to FEniCS (https://fenicsproject.org, accessed on 5 April 2025)  [38,39], an open-source computing platform for solving partial differential equations (PDEs).

## 5. Numerical Results

This section presentes the numerical results obtained by solving the problem described in Section 2 using the numerical implementation detailed in Section 4. Starting from the analytical solution discussed in Section 3, this study explores two different scenarios: (i) the transformation from an auxetic to a non-auxetic mechanical behavior of a material in class (A) under specific initial fiber orientation conditions, and (ii) the segregation process of the fiber orientation field which is characteristic of materials in class (C). Materials in class (B) are not considered in this work, as they do not exhibit any extreme behavior. Once perturbed, fibers in class (B) materials tend to reorient monotonically toward a single stable configuration, making their evolution relatively straightforward compared to the bistable or metastable behaviors of classes (A) and (C). However, such materials are particularly relevant in applications where a predictable and stable anisotropic response is desired, such as tendon-like biological tissues or fiber-reinforced composites designed for unidirectional strength. For further details about this material, the reader is referred to [22].

### 5.1. Auxetic to Non-Auxetic Trasformation

**Figure 4:**
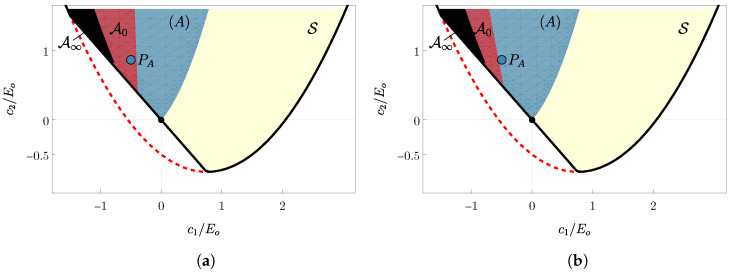
The black region represents the set of anisotropic coefficients that exhibit auxetic behavior at the asymptotic orientation $\vartheta_{\infty }$, denoted as $A_{\infty }$. The red region represents the auxetic set $A_{0}$. The panel (**a**) shows the latter set with $\vartheta_{0}=6$°, while the panel (**b**) shows the same set with $\vartheta_{0}=\vartheta_{s1}^{A}≃15$°.

The present section focuses on a specific material in class (A), represented by the blue dot, $P_{A}$, in Figure 1b and Figure 4. This material has anisotropic parameters $c_{1}=-0.75$ and $c_{2}=1.3$, while the Lamé coefficients are chosen as λ=μ=3/8. Equation (13) provides the asymptotic orientation, which is $\vartheta_{\infty }≃54$°. As seen in Section 3, $\vartheta_{\infty }$ is a minimum of the free energy and coincides with the limit orientation that the fibers will tend towards during the loading process, regardless of the initial orientation.

In Figure 4, two similar schematics are shown where the black region represents the set of anisotropic coefficients that exhibit auxetic behavior at $\vartheta_{\infty }$, denoted as $A_{\infty }=\left\{\left(c_{1},c_{2}\right)\in S:\nu \left(\vartheta_{\infty }\right)<0\right\}$. The material under considerations, represented by point $P_{A}$, does not belong to this area, indicating that the asymptotic orientation does not exhibit auxetic behavior. Conversely, the red region represents the auxetic set $A_{0}=\left\{\left(c_{1},c_{2}\right)\in S:\nu \left(\vartheta_{0}\right)<0\right\}$. In panel (a), $\vartheta_{0}=6$°$<\vartheta_{s1}^{A}$, while in panel (b), $\vartheta_{0}=\vartheta_{s1}^{A}≃15$°. Although the black regions are identical in both panels, the red areas differ. On the left, $P_{A}$ belongs to $A_{0}$, while on the right, it does not. This indicates that the material $P_{A}$ is a good candidate to perform the transition from auxetic to non-auxetic: with an initial orientation $\vartheta_{0}=6$°, it behaves like an auxetic material, but when the fiber reaches its limit orientation, the material no longer exhibits auxetic behavior.

**Figure 5:**
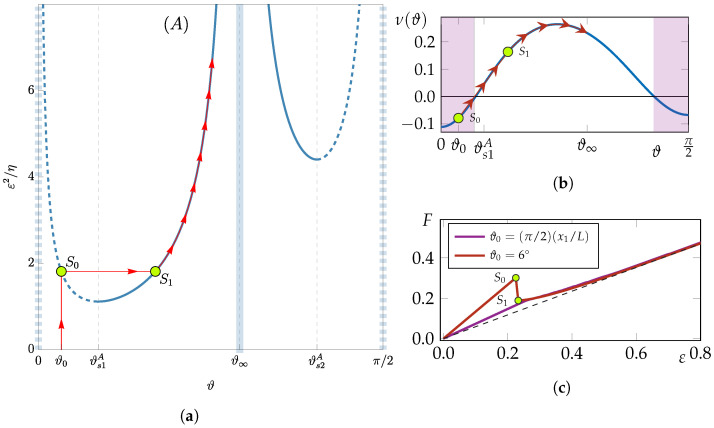
(**a**) The reorientation path in the plane $\left\{\vartheta ,\varepsilon^{2}/\eta \right\}$ is represented by the continuous red line with arrows. (**b**) Poisson ratio landscape for the material in class (A) with $c_{1}=-0.75$ and $c_{2}=1.3$. (**c**) Force *F* as a function of the applied displacement ε.

**Figure 6:**
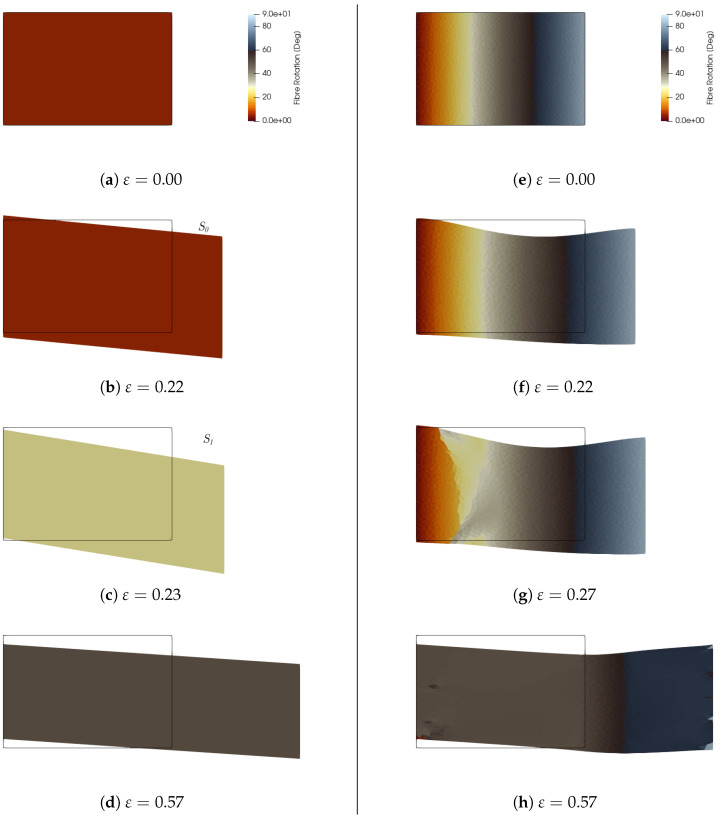
Auxetic to non-auxetic transition. Material in class (A) with $c_{1}=-0.75$ and $c_{2}=1.3$. (**a**–**d**) Left panel from top to bottom: (**a**) initial undeformed configuration with constant distribution of $\vartheta_{0}=6$° followed by (**b**) the auxetic behavior, (**c**) the transition to non-auxetic, and finally (**d**) the asymptotic value $\vartheta_{\infty }≃54$°. (**e**–**h**) Right panel from top to bottom: (**e**) initial undeformed configuration with linear distribution of $\vartheta_{0}=\pi /2x_{1}/L$ followed by (**f**) the simultaneous auxetic (sides) and non-auxetic (middle) behaviors, (**g**) the propagation of the non-auxetic phase, and finally (**h**) reaching the limit value almost everywhere $\vartheta_{\infty }≃54$°.

In Figure 5a, the reorientation path is represented by red lines with arrows as ε increases, while Figure 5b shows the complete landscape of the Poisson ratio, given by Equation (4). Figure 6a–d presents four snapshots taken along this loading path: the snapshot (a) shows the initial orientation and the undeformed shape of the sample. As ε increases, the homogeneous material reaches the boundary of the reversibility domain, labeled $S_{0}$ in Figure 5. The behavior is auxetic, as seen in the deformed shape in Figure 6b snapshot $S_{0}$, where, for instance, the left side of the deformed shape is larger than the same side of the original undeformed shape (black rectangle). Upon reaching this limit, the orientation evolves, with ϑ jumping from $\vartheta_{0}=6$° to 30°, labeled $S_{1}$ in Figure 6c. From this snapshot and the Poisson ratio landscape, one can observe that the behavior is no longer auxetic until it reaches the asymptotic value of 54°, Figure 6d.

The same process is analyzed for a material in class (A) with a linear distribution of the initial fiber orientation, $\vartheta_{0}=\left(\pi /2\right)\left(x_{1}/L\right)$, as shown in the right panel of Figure 6e–h. At small load levels ε, the left and right extremities of the deformed shape exhibit auxetic behavior, characterized by an increase in size, as indicated by the violet region in the Poisson ratio landscape. As the loading progresses, the material transitions towards the asymptotic orientation $\vartheta_{\infty }≃54$°, which is nearly achieved everywhere in the configuration shown in Figure 6h. However, it is important to note that the exact asymptotic value is only fully reached as ε→∞, as depicted in Figure 5a. Additionally, the yield set for fibers oriented at $\vartheta >\vartheta_{\infty }$ is reached at higher values of ε compared to fibers oriented at $\vartheta <\vartheta_{\infty }$.

Finally, in Figure 5c, the material response is shown in terms of force *F* as a function of the applied displacement ε. The force is computed at each load step as the integral of the traction vector $Tn_{\Gamma }$ over the boundary, $\partial_{u}B$, where the displacement is applied:

(16) $$F=\int_{\partial_{u}B}Tn_{\Gamma }\;d\Gamma ,$$

where $n_{\Gamma }$ is the normal unit vector to that boundary. In this panel, the red line represents the response of the material with initial orientation $\vartheta_{0}=6$°, with the drop $S_{0}$–$S_{1}$ indicating the sudden reorientation. The purple line represents the response of the material with a linear distribution of the initial orientation $\vartheta_{0}=\left(\pi /2\right)\left(x_{1}/L\right)$. Both responses tend towards the same asymptotic behavior at large ε, represented by the dashed black line.

### 5.2. Segregation Process

To illustrate the segregation process, the analysis focuses on a material from class (C), represented by the red dot $P_{C}$ in Figure 1b. This material has anisotropic parameters $c_{1}=1.5$ and $c_{2}=0$, while the Lamé coefficients are chosen as λ=μ=3/8. The asymptotic orientation is $\vartheta_{\infty }≃30$°. For this specific material class (C), $\vartheta_{\infty }$ becomes the maximum of the free energy and corresponds to the critical point, which means that a fiber initially oriented as $\vartheta_{\infty }\pm \alpha$, with α a small perturbation, will reorient towards π/2 or 0, respectively. This section presents two study cases, in which the author explores the evolutions of different non-uniform distributions of initial orientation $\vartheta_{0}\left(x\right)$. The first one has a piece-wise linear distribution along the coordinate $x_{1}$:

(17) $$\vartheta_{0}^{i}\left(x_{1}\right)=\left\{\begin{array}{cc} \frac{\pi }{5L}x_{1} & \mathrm{if}\;0\leq x_{1}\leq \frac{L}{2},\\ \frac{\pi }{2L}x_{1} & \mathrm{if}\;\frac{L}{2}>x_{1}>L. \end{array}\right.$$

**Figure 7:**
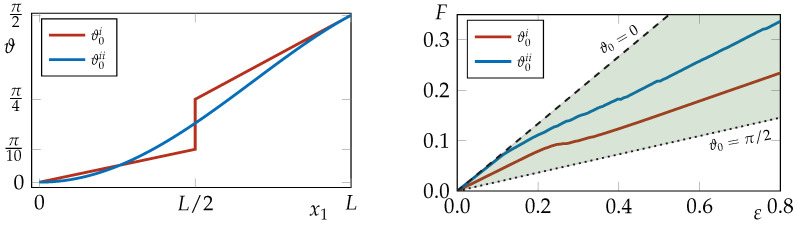
(Left) Initial distributions of the fiber orientation for cases: (i) $\vartheta_{0}^{i}$ Equation (17) and (ii) $\vartheta_{0}^{ii}$ Equation (18) both plotted along the dashed white line in Figure 8 top row. (Right) Force *F* as a function of the applied displacement ε.

**Figure 8:**
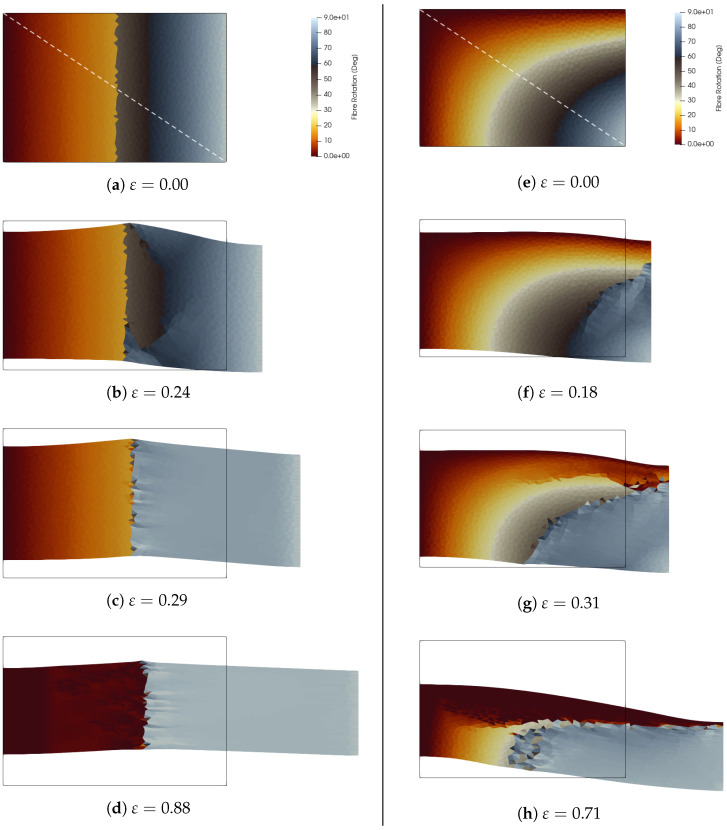
Segregation process. Material in class (C) with $c_{1}=1.5$ and $c_{2}=0$. (**a**–**d**) Left panel from top to bottom: (**a**) initial undeformed configuration with piece-wise linear distribution of $\vartheta_{0}^{i}$ given by Equation (17), (**b**) the segregation front on the right half of the sample, (**c**) the completely segregated half sample, and (**d**) the final distribution: the red part is at 0° while the grey half has an orientation equal to 90°. (**e**–**h**) Right panel from top to bottom: (**e**) initial undeformed configuration with distribution of $\vartheta_{0}^{ii}$ given by Equation (18), (**f**) the segregation process is activated from the bottom right corner, in (**g**) the segregation front starts to appear, and (**h**) reports the final segregated distribution.

This is also plotted in red in the left panel of Figure 7 along the sample diagonal, and is shown by the white line in Figure 8a.

The second study case has an initial distribution that follows the function:

(18) $$\vartheta_{0}^{ii}\left(x\right)=\frac{\pi }{2L}x_{1}\cos\left(\frac{\pi }{2H}x_{2}\right)\;\mathrm{with}\;x_{1}\in \left[0,L\right]\;\mathrm{and}\;x_{2}\in \left[0,H\right],$$

this becomes 0 along the left and top edges and reaches π/2 in the bottom-right corner $x_{1}=L$ and $x_{2}=0$; see the contour plot of snapshot on the panel (e) in Figure 8 and blue line in the left panel of Figure 7 for the effective distribution along the diagonal of the sample.

The evolution of the fiber orientation for these two cases is depicted in Figure 8. The left panel shows the deformed shape and the evolution, as a contour plot, of the fiber orientation field for the piece-wise linear distribution given by Equation (17), $\vartheta_{0}^{i}$. The right panel shows the deformation and reorientation for $\vartheta_{0}^{ii}$, given by Equation (18). The top row of Figure 8 shows the initial distribution of the fiber orientation, while the bottom row shows the final distribution. In both cases, the segregation process is evident, particularly in the second study case where one can observe the propagating front of segregation. The final distributions show fibers reaching the two minima at 0° and 90°, polarizing the sample in two regions.

Finally, the force–displacement curve is shown in the right panel of Figure 7. The red line represents the response of the material with the piece-wise linear distribution of the initial orientation, while the purple line represents the response of the material with the distribution (18). Both responses are confined within the green cone, which is bounded by the responses of the material characterized by initial fiber orientations of 0° (dashed black line) and 90° (dotted black line).

## 6. Practical Implications

One key aspect of the results is the emergence of phase segregation, where distinct fiber orientations develop within different regions of the material. This phenomenon leads to localized variations in mechanical properties, potentially influencing failure mechanisms, toughness, and adaptability. In engineering applications, such behavior can be leveraged to design materials with tailored mechanical responses, such as controlled deformation patterns or enhanced energy dissipation. In biological tissues, phase segregation may play a role in structural adaptation processes, allowing different regions to develop specialized mechanical functions over time [40,41].

Furthermore, the transition from auxetic to non-auxetic behavior, governed by fiber alignment, offers opportunities for designing materials with tunable mechanical properties. Auxetic materials, characterized by their negative Poisson ratio, are known for their enhanced fracture resistance and superior shock absorption [42,43]. A deeper understanding of this transition can facilitate the development of smart materials for applications in biomedical implants, soft robotics, and impact-resistant structures.

By capturing these extreme behaviors, this study lays the foundation for designing advanced fibrous materials with programmable mechanical properties. The findings contribute to optimizing composite structures, improving the durability of biomimetic materials, and refining computational models for predicting material responses under various loading conditions. Moreover, the theoretical framework and numerical simulations presented here provide a basis for future experimental validation and practical implementation in real-world applications.

## 7. Concluding Remarks and Future Perspectives

This work presents a comprehensive continuum model for fibrous materials undergoing plastic remodeling, leading to significant changes in fiber orientation and, consequently, their mechanical response. The study demonstrates that such remodeling processes can result in complex material behaviors, including phase segregation and transitions between auxetic and non-auxetic properties, with far-reaching implications for both engineered materials and biological tissues.

Through a combination of analytical and numerical approaches, the author demonstrates how the reorientation of fibers can drastically alter the mechanical response of the material. Specifically, three distinct classes of material behavior were identified based on the peculiarities of the free energy function: materials that exhibit a stable fiber orientation at an intermediate angle, materials that align fibers perpendicularly to the load direction, and materials that can exhibit either behavior depending on the initial fiber orientation. This classification underscores the crucial role of fiber orientation in governing the mechanical properties of fibrous materials.

Beyond theoretical insights, the findings have practical relevance for material design and optimization. The emergence of phase segregation—where different regions of the material develop distinct fiber orientations—has implications for localized mechanical properties, influencing structural toughness, failure mechanisms, and adaptability. This phenomenon could be exploited to engineer materials with controlled deformation patterns, improved energy dissipation, or enhanced durability in applications ranging from soft robotics to impact-resistant structures. Similarly, the ability to transition between auxetic and non-auxetic behaviors offers exciting possibilities for tunable materials. Auxetic structures, known for their enhanced fracture resistance and shock absorption, could be tailored for biomedical implants, protective equipment, and morphing materials capable of adjusting their mechanical response in real time.

Numerical simulations have provided further insights into the evolution of fiber orientation under applied loads. These insights pave the way for the development of fibrous materials with programmable mechanical properties, optimizing composite structures and biomimetic materials for specialized applications.

Future work will address the current limitations by extending the model to three-dimensional settings, enabling a more comprehensive understanding of remodeling in real-world scenarios. Additionally, incorporating viscous effects will enhance the model’sability to capture time-dependent material responses, improving its predictive accuracy and facilitating closer alignment with experimental observations.

Overall, this study lays a solid foundation for advancing the understanding of fibrous materials undergoing plastic remodeling, offering new opportunities for the design of materials with tailored mechanical properties for engineering, biomedical, and industrial applications.   

## Figures and Tables

**Table 1:** Notations used in the model.

Symbol	Description
x	Position vector of a point in the body.
ϑ	Internal state variable representing fiber orientation.
E	Linearized strain tensor, based on displacement field u.
Λ	State of the body at time *t*, consisting of strain E and fiber orientation ϑ.
n	Unit vector in the fiber direction, depending on the internal state variable ϑ.
C	Elasticity tensor for transversely isotropic material in the fiber direction n(ϑ).
μ	Lamé coefficient (shear modulus).
λ	Lamé coefficient (first Lamé constant).
$c_{1}$	Material constant describing transverse isotropy.
$c_{2}$	Material constant describing transverse isotropy.
*E*	Young’s modulus.
ν	Poisson ratio.
γ	Remodeling torque, associated with changes in fiber orientation ϑ.
$\bar{\eta }$	Dissipation potential, associated with fiber reorientation.
$\bar{\Theta }$	Accumulated fiber rotation over time *t*.
ψ	Free energy density, a function of E and ϑ.
δ	Remodeling dissipation.
E	Free energy density, a function of E and ϑ.
P	Set of valid pairs $\left(c_{1},c_{2}\right)$ ensuring positive free energy density.
S	Stiffening set, representing materials where fibers align to enhance stiffness.
C	Compliance set, representing materials where fibers reduce stiffness in their orientation direction.
R	Reversibility domain where the remodeling torque is below a critical threshold.
∂R	Boundary of the reversibility domain: yield set.

## Data Availability

No data were used to support this study.

## Appendix A

In this appendix, the author reports the algorithm presented in Section 4, which outlines the alternating minimization scheme used to solve the coupled problem of displacement and fiber remodeling in fibrous materials.

**Algorithm A1** Alternating Minimization Scheme
**Require:** Initial values $\left\{u_{0},\vartheta_{0},{\bar{\Theta }}_{0}\right\}$ and Dirichlet boundary conditions **Ensure:** Solution fields $\left\{u_{n},\vartheta_{n},{\bar{\Theta }}_{n}\right\}$ at the final time step 1: Initialize: i=0, $u_{0}=0$, $\vartheta_{0}=0$, ${\bar{\Theta }}_{0}=0$ 2: **for** i=1,2,…,n **do** 3: Update Dirichlet boundary conditions based on the current time step 4: **while** $u_{err}>u_{tol}$ AND $\vartheta_{err}>\vartheta_{tol}$ **do** 5: Solve the linear elastic problem for $u_{i}$: 6: $u_{i}=\underset{u}{\arg\min}\left\{E_{i}\left(u,\vartheta_{i-1};{\bar{\Theta }}_{i-1}\right)\right\}$ 7: Evaluate the yield function $\gamma (u_{i},\vartheta_{i-1})$ 8: **if** $\gamma \left(u_{i},\vartheta_{i-1}\right)\leq \bar{\eta }$ **then** 9: Remodeling does not occur; $\vartheta_{i}=\vartheta_{i-1}$ 10: **else** 11: Initialize local projection iteration: j=0, $\vartheta_{i}^{0}=\vartheta_{i-1}$ 12: **while** $\vartheta_{err,proj}>\vartheta_{tol,proj}$ **do** 13: Increment projection iteration: j=j+1 14: Solve the remodeling problem via local projection: 15: $\vartheta_{i}^{j}=\mathrm{Projection}\left(\vartheta_{i}^{j-1}+\Delta \vartheta^{j}\right)$ 16: Update projection error: $\vartheta_{err,proj}={∥\vartheta_{i}^{j}-\vartheta_{i}^{j-1}∥}_{L_{2}}$ 17: **end while** 18: $\vartheta_{i}=\vartheta_{i}^{j}$ 19: **end if** 20: Update displacement error: $u_{err}={∥u_{i}-u_{i-1}∥}_{H_{1}}$ 21: Update fiber rotation error: $\vartheta_{err}={∥\vartheta_{i}-\vartheta_{i-1}∥}_{L_{2}}$ 22: **end while** 23: Update accumulated fiber rotation: ${\bar{\Theta }}_{i}={\bar{\Theta }}_{i-1}+∥\vartheta_{i}+\vartheta_{i-1}∥$ 24: **end for**
